# Bilayer-Forming Lipids Enhance Archaeal Monolayer Membrane Stability

**DOI:** 10.3390/ijms26073045

**Published:** 2025-03-26

**Authors:** Margot Saracco, Philippe Schaeffer, Maxime Tourte, Sonja-Verena Albers, Yoann Louis, Judith Peters, Bruno Demé, Stephane Fontanay, Philippe M. Oger

**Affiliations:** 1 INSA Lyon, Universite Claude Bernard Lyon 1, CNRS UMR5240, F-69100 Villeurbanne, France; margot.saracco@insa-lyon.fr (M.S.); yoann.louis@insa-lyon.fr (Y.L.); stephane.fontanay@insa-lyon.fr (S.F.); 2 Biogéochimie Moléculaire, University of Strasbourg, CNRS UMR 7177, F-67000 Strasbourg, France; p.schaef@unistra.fr; 3 Molecular Biology of Archaea, Institute of Biology, University of Freiburg, D-79104 Freiburg, Germany; maxime.tourte@gmail.com (M.T.); sonja.albers@biologie.uni-freiburg.de (S.-V.A.); 4 Institut Laue Langevin, F-38042 Grenoble, France; peters@ill.fr (J.P.); deme@ill.fr (B.D.); 5 Interdisciplinary Laboratory of Physics, Université Grenoble Alpes, CNRS UMR5588, F-38400 Grenoble, France; 6 Institut Universitaire de France, F-75231 Paris, France

**Keywords:** Archaea, monolayer membrane, tetraether, archaeal lipids, temperature stability, neutron diffraction, membrane biophysics

## Abstract

Archaeal membranes exhibit remarkable stability under extreme environmental conditions, a feature attributed to their unique lipid composition. While it is widely accepted that tetraether lipids confer structural integrity by forming monolayers, the role of bilayer-forming diether lipids in membrane stability remains unclear. Here, we demonstrate that incorporating diethers into archaeal-like lipid assemblies enhances membrane organization and adaptability under thermal stress. Using neutron diffraction, we show that membranes composed of mixed diethers and tetraethers exhibit greater structural order and stability compared to pure lipid systems. Contrary to expectations, monolayer-forming tetraethers alone display increased variability in lamellar spacing under fluctuating temperature and humidity, whereas mixed lipid membranes maintain a consistent architecture. Furthermore, neutron-scattering length density profiles reveal an unexpected density feature at the bilayer midplane, challenging conventional models of archaeal monolayer organization. These findings suggest that molecular diversity of lipid molecules, rather than tetraether dominance, plays a critical role in membrane auto-assembly, stability, and adaptability. Our results provide new insights into archaeal membrane adaptation strategies, with implications for the development of bioinspired, robust synthetic membranes for industrial and biomedical applications.

## 1. Introduction

All living cells synthesize one or more plasma membranes, which isolate the intracellular space from the external environment. Acting as a diffusion barrier for ions and small solutes, the membrane is essential not only for maintaining cellular integrity but also as a support for numerous cellular activities. This dynamic structure controls cell shape and growth, mediates vesicle release and integration, and facilitates intercellular communication [1,2]. Maintaining membrane integrity and physico-chemical parameters is, therefore, critical for cells. Early works have set the base for the concept of homeoviscous adaptation [3,4], which states cells will maintain the viscosity of the membrane when growth conditions vary. Membrane integrity is continuously secured dynamically through modifications of their plasma membrane lipid composition to maintain optimal fluidity and functionality. With the evolution of the knowledge of membrane structure, this concept has evolved. Nowadays, it refers to the maintenance of all membrane parameters (viscosity, rigidity, inward and outward permeability to water, proton and other molecules, protein docking, etc.). In Bacteria and Eukarya, homeoviscous adaptation primarily involves varying the acyl chain length and/or the number of unsaturations along the acyl chain or modifying the proportions of the different polar headgroups [5,6].

Among the three domains of life, Archaea are distinguished by the unique composition of their membrane lipids, setting them apart from Bacteria and Eukarya. Archaeal lipids are characterized by two polyisoprenoid alkyl chains linked to glycerol via ether bonds in a *sn*-2,3 configuration [7,8]. In contrast, bacterial and eukaryotic membrane lipids comprise two fatty acyl chains ester-linked in a *sn*-1,2 configuration [9]. These distinct features are commonly acknowledged as key components of archaeal membrane adaptations, enabling them to thrive in extreme environments. The ether linkages in archaeal membranes are more chemically and thermally resistant than the ester linkages in bacterial and eukaryotic membranes [10,11]. Furthermore, the ether bonds in the hydrophobic core promote tighter packing of the polar headgroups, increasing water impermeability and reducing electrostatic interactions [10,11]. In addition, the archaeal isoprenoid hydrocarbon chains with branched methyl groups enhance lipid packing and membrane rigidity compared to the linear acyl chains generally found in bacterial and eukaryotic lipids. These adaptations result in increased stability and impermeability under extreme conditions [12,13]. In addition, Archaea also synthesize monolayer-forming tetraether lipids, which consist of two transmembrane C_40_ isoprenoid side chains ether-linked to two glycerol moieties [14]. Unlike conventional lipids, tetraether lipids can form a monolayer membrane [15], increasing lipid packing density [16,17] and reducing proton permeability [12]. The structural diversity of tetraether lipids includes glycerol mono-, di-, and trialkyl glycerol tetraethers (GMGT, GDGT, and GTGT, respectively) [18], with unsaturated/cyclized, hydroxylated, or methylated isoprenoid chains [19]. Tetraether lipids may also contain up to eight cyclopentane rings and/or one cyclohexane ring [20]. Since these bipolar lipids exhibit unique behaviors, forming membranes that are more stable, rigid, and impermeable than those of Bacteria or Eukarya, it has been largely admitted that the synthesis of bi-polar, tetraether, monolayer-forming lipids is a necessary adaptation for life at high temperatures [6,21].

In Archaea, the concept of homeoviscous adaptation is also valid, despite the difference in lipid structures, confirming the necessity for all types of cells to maintain their membrane functionality. It involves mechanisms similar to those of Bacteria and Eukarya, e.g., the variation in the number of unsaturation of the hydrophobic chains and possibly the variation in the proportion of the polar headgroups [6,22]. It also involves Archaea-specific mechanisms, such as the variation in the proportion of tetraether lipids in the membrane [22,23,24]. However, despite the long-standing belief that tetraethers are necessary for heat tolerance, recent findings suggest a more complex picture. Several hyperthermophilic Archaea, including *Methanopyrus kandleri* and *Aeropyrum pernix*, exhibit little to no tetraether lipids yet grow optimally at 95–105 °C (Table 1) [25,26]. Similarly, in *Thermococcus kodakarensis*, the deletion of the tetraether synthase (Tes) enzyme did not prevent growth at 95 °C, demonstrating that tetraether biosynthesis is not essential for high-temperature survival [27]. Furthermore, numerous hyperthermophilic Archaea, such as members of the class Thermococci, which are able to grow up to 113 °C, harbor a mixture of diether and tetraether lipids. In these hyperthermophilic Archaea that produce a mixture of diether and tetraether lipids, the proportion of diether lipids in the membrane is still significant even at the highest growth temperature.

These observations challenge our understanding of archaeal membrane adaptation to extreme temperatures. Studying the homeoviscous adaptation in the hyperthermophilic *Thermococcus barophilus*, Cario and colleagues have shown, as expected, that the homeoviscous adaptation involves the fine regulation of the ratio of diether and tetraethers [23]. However, they have also shown the dominance of diether lipids in its membranes even at 100 °C, suggesting that in *T. barophilus*, membrane domains of monolayer and bilayer may co-exist due to lipid phase separation [28,29,30]. To explain the high-temperature stability of the bilayer in this species, they have proposed a novel membrane ultrastructure, which involves the presence of non-polar polyisoprenoids in the midplane of the bilayer [23]. Experimental studies using synthetic archaeal lipid analogs have demonstrated the validity of this ultrastructure, the presence of the apolar isoprenoids in the bilayer midplane, and the impact on membrane properties, e.g., increased rigidity, decreased permeability to protons, and impact on the bending ability of the membrane [31], which are congruent with the observed homeoviscous response observed in *T. barophilus*.

**Table 1:** Comparison of the diether/tetraether ratio for different archaeal species as a function of the optimum growth temperature. D = diether and T = tetraether, including only GDGT lipids. Species in bold correspond to the species used in this study. The dotted line separates mesophilic from hyperthermophilic species. ND = not detected.

Species	Order	Kingdom	Optimal Growth Temperature (°C)	Lipids (%)		References
				D	T	
*N. piranensis* (*C*)	Thaumarchaeota (Nitrosopumilales)	Proteoarchaeota	25	8	68	[32]
*M. boonei*	Stenosarchaea (Methanomicrobiales)	Euryarchaeota	35	4	96	[33]
*M. concilii*	Stenosarchaea (Methanosarcinales)	Euryarchaeota	35	70	ND	[34]
*T. acidophilum*	Thermoplasmatales	Euryarchaeota	55–59	90	10	[35]
*M. Prunae*	Crenarchaeota (Sulfolobales)	Proteoarchaeota	75	Traces	70	[36]
***S. acidocaldarius***	**Crenarchaeota (Sulfolobales)**	**Proteoarchaeota**	**80**	**25**	**75**	[37]
*T. kodakarensis*	Thermococcales	Euryarchaeota	85	41	59	[21]
*T. barophilus MP*	Thermococcales	Euryarchaeota	85	55.1	44.3	[38]
*A. pernix*	Desulfurococcales	Proteoarchaeota	90–95	100	0	[39]
*I. aggregans*	Thermoproteota (Desulfurococcales)	Proteoarchaeota	93.5	Traces	61	[18]
*M. kandleri*	Methanopyrales	Euryarchaeota	98	100	0	[25]
***P. furiosus***	**Thermococcales**	**Euryarchaeota**	**100**	**30**	**50**	[40]

To date, the questions of the spatial distribution of lipids in a membrane composed of a mixture of diether and tetraether lipids and the physicochemical properties of such a mixed lipid membrane remain open. Indeed, it is unclear whether the two types of lipids will mix homogeneously or separate in different phases as observed for pure lipid [28,29,30], due to the difference in length of their hydrophobic core and whether the mixture will harbor improved or degraded properties in comparison to membranes composed of pure diether or tetraether lipids.

We hypothesize that tetraether-rich membranes will exhibit enhanced stability compared to diether-rich membranes and that incorporating tetraethers into bilayer membranes should reinforce membrane integrity at high temperatures. To test this hypothesis, we investigated the structural behavior of reconstructed archaeal membranes containing varying concentrations of tetraether lipids by neutron diffraction under conditions of thermal stress. Contrary to expectations, our findings revealed that membranes composed of pure tetraether lipids were less stable than membranes composed of a mixture of di- and tetraether lipids. This suggests that membrane resilience benefits from lipid diversity, with a combination of flexible diethers and rigid tetraethers providing an optimal balance for structural integrity.

To elucidate these observations and assess the necessity of tetraethers for membrane stability at high temperatures, we investigated the behavior of archaeal-like bilayer membranes containing varying concentrations of tetraethers. Using purified lipids from *Pyrococcus furiosus* and *Sulfolobus acidocaldarius*, we conducted neutron diffraction experiments under high-temperature conditions to mimic the extreme environments inhabited by Archaea. This approach allowed us to characterize the ultrastructural response of the membranes to temperature variations as a function of tetraether concentration. It also provided crucial insights into how membrane architecture adapts to thermal stress, shedding light on the fundamental principles of cellular resilience. Understanding these mechanisms not only deepens our knowledge of archaeal survival strategies but also offers potential applications in biotechnology, such as engineering heat-resistant biomaterials and developing robust synthetic membranes for industrial processes.

## 2. Results

### 2.1. Purified Lipid Characterization

**Figure 1:**
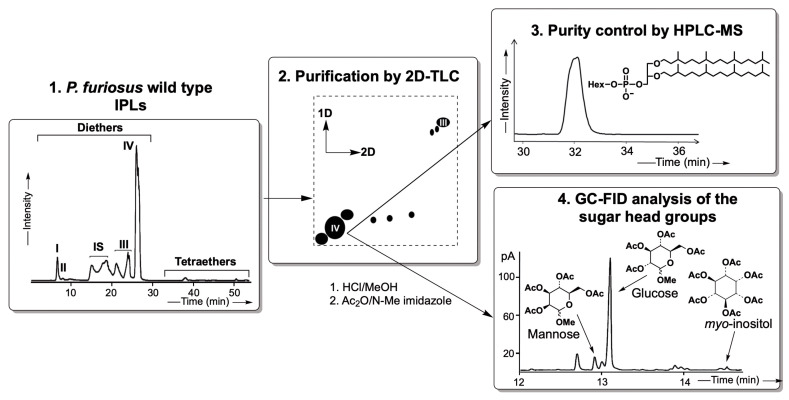
Schematic representation of PI-DGD purification steps and identification of the hexose head groups. (**1**) HPLC-ESI-MS (negative ions) base peak chromatogram of IPLs of *P. furiosus* wild-type strain; I: P-DGD; II: PG-DGD; III: PHexNHAc-DGD; IV: PHex-DGD. (**2**) Purification of PHex-DGD by two-dimensional thin layer chromatography (1D = (TCM/MeOH/H_2_O; 75:25:2.5; *v*/*v*/*v*); 2D = (TCM/MeOH/AcOH/H_2_O; 80:9:12:2; *v*/*v*/*v*/*v*) [43]. (**3**) HPLC-ESI-MS (negative ions) base peak chromatogram for controlling the purity of the isolated compound PHex-DGD. (**4**) Methanolysis of the purified PHex-DGD followed by acetylation and GC-FID analysis to identify the hexose head groups.

The diether lipid (D) used in our experiments consisted of a dialkylglycerol diether (DGD) with a phosphatidyl hexose polar headgroup (PHex-DGD; [M-H]^−^ 893) (Figure 1(3)). Following extraction and purification from *Pyrococcus furiosus* lipid extracts, acid hydrolysis and acetylation of an aliquot of the isolated PHex-DGD revealed that the polar headgroup was a mixture of methoxy sugars, primarily glucose (89%), along with mannose (9%) and inositol (2%) (Figure 1(4)). The diether could not be further purified due to the similar polarity of these hexose polar headgroups on the silica gel used to separate them by 2D thin-layer chromatography or HPLC-MS. These stereoisomers elute simultaneously and exhibit identical masses, making individual separation challenging [41,42].

**Figure 2:**
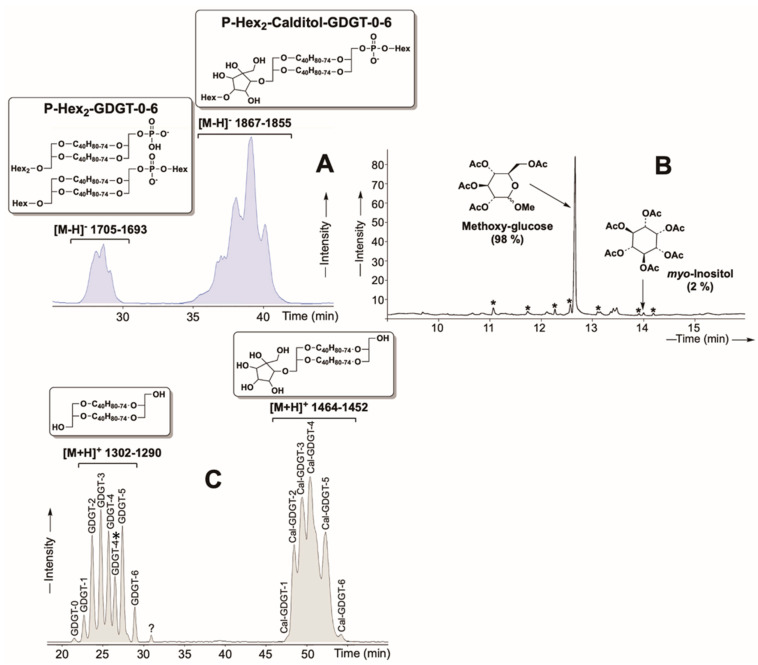
Characterization of the tetraether mixture isolated from the lipid extract from *S. acidocaldarius*. (**A**) HPLC-ESI-MS (negative ions) base peak chromatogram of the tetraether polar lipid fraction isolated from *S. acidocaldarius.* (**B**) Gas chromatogram (GC-FID) showing the sugar head groups recovered upon methanolysis of the isolated tetraether mixture from *S. acidocaldarius*. The sugars are analyzed as acetate derivatives. *: contaminations. (**C**) HPLC-APCI-MS (positive ions) base peak chromatogram of the core lipids recovered upon methanolysis of the tetraether polar lipid fraction isolated from *S. acidocaldarius*. GDGT-0-6: Glycerol dialkyl glycerol tetraether with 0 to 6 cyclopentane rings. GDGT-4* = structural isomer of GDGT-4. Cal-GDGT-0-6: Calditol-GDGT with 0 to 6 cyclopentane rings.

The tetraether lipids (T) were purified from the polar lipid fraction E of *Sulfolobus acidocaldarius* [44]. To obtain pure tetraethers, we developed a novel lipid separation approach involving protection and deprotection steps (Schaeffer et al., in preparation. See details in the Supplementary Materials). Despite multiple purification attempts, complete separation of individual tetraether molecules was not achieved. The final purified extract consisted of two major glycerol dialkylglycerol tetraether (GDGT) series, namely P-Hex_2_-GDGT-0 to 6 ([M-H]^−^ 1705–1693 Da) and P-Hex_2_-Calditol-GDGT-0 to 6 ([M-H]^−^ 1867–1855 Da) (Figure 2A,C). Both series contained two C_40_ isoprenoid (biphytane) chains with 0 to 6 cyclopentane rings (Figure 2C). Acid hydrolysis followed by acetylation of this tetraether mixture revealed that the hexose groups were predominantly glucose (98%), with a minor fraction of inositol (2%) (Figure 2B).

### 2.2. Membrane Ordering and Stability Across Experimental Conditions

**Figure 3:**
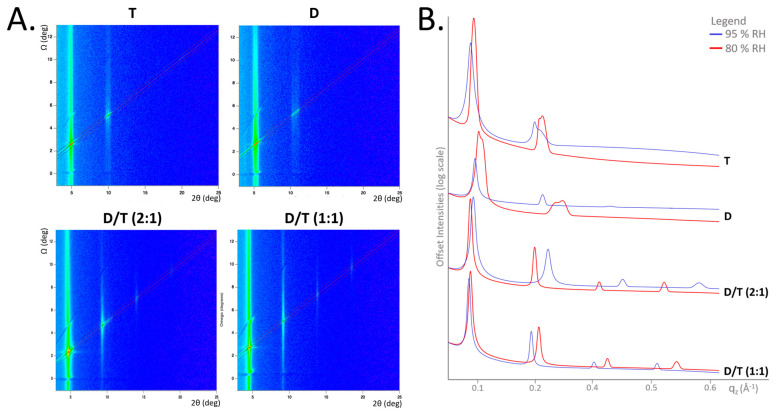
Neutron diffraction of multilayer samples with varying concentrations of purified tetraether (T) and diether lipids (D). (**A**) Reciprocal space maps resulting from typical Ω-scans/(2θ, Ω) showing the Bragg peak positions of the different lipid samples, run at 100% D_2_O 70 °C 80% RH. (**B**) 1D integrated intensity along the Z direction of the different lipid samples, highlighting Bragg peak intensities, run at 100% D_2_O 70 °C. Red lines represent 80% RH measurements, and blue lines 95% RH measurements. D = diether sample, T = tetraether sample, D/T = mixture of diether and tetraether at different molar ratios (1:1) or (2:1).

The ability of our natural lipids to self-assemble into oriented multilayer structures was confirmed using neutron diffraction. Typically, lipid films hydrated by water vapor form stacks of bilayers separated by interstitial water layers, leading to the appearance of Bragg peaks in the diffraction data. In our study, the stacked multilayers were sufficiently well-ordered to produce Bragg peaks across all tested conditions for the four lipid samples (Figure 3A).

Lipid composition had a significant influence on membrane organization and stability. Membranes composed solely of diethers or tetraethers exhibited broader, less-defined Bragg peaks, whereas those containing a mixture of both lipids (D/T mixtures) displayed sharper, more intense peaks. In the 1D neutron diffractogram, D/T mixtures showed four well-defined Bragg peaks, indicating higher structural organization, while pure diether and tetraether membranes exhibited broader peaks at both 80% and 95% relative humidity (RH) (Figure 3B). At 80% RH, broad scattering patterns in T and D membranes suggested structural inhomogeneity, likely due to the coexistence of multiple lamellar phases.

At 95% RH, the diether membrane reorganized into a single lamellar phase with three Bragg orders, while the tetraether sample remained somewhat inhomogeneous yet still maintained three Bragg peaks at elevated temperatures (Figure 3B). Notably, D/T mixtures consistently exhibited strong signals with four Bragg peaks under all conditions, even at high temperatures of 80 °C and 90 °C, which represents the operational limit of the BerILL setup (Figure S1).

### 2.3. d-Spacing Fluctuation Across Temperature and Humidity

**Figure 4:**
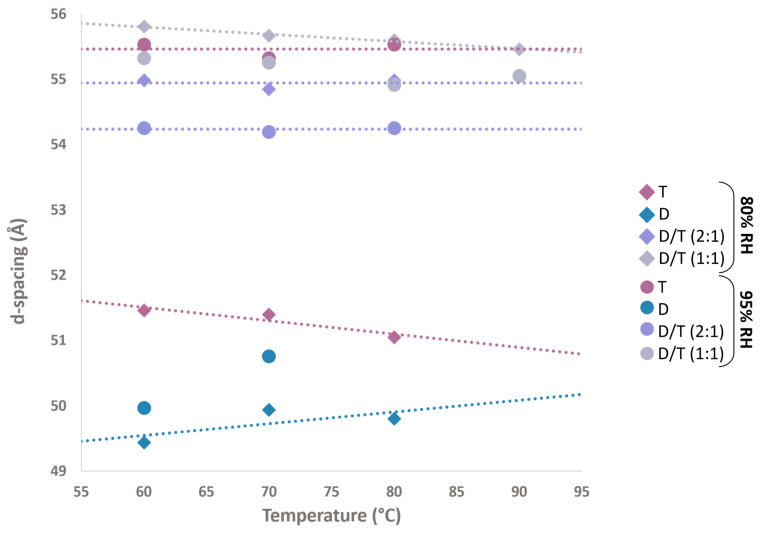
Lamellar d-spacing as a function of temperature and relative humidity for different lipid compositions. Measured at 80% and 95% RH at 60 °C, 70 °C, and 80 °C. Only one sample was measured at 90 °C. The d-spacing was calculated only from 8% D_2_O measurements (see methods). D = diether sample, T = tetraether sample, D/T = mixture of diether and tetraether polar lipids at different molar ratios (1:1) and (2:1). A problem occurred during the diether measurement at 80 °C 95% RH, and it was thus not possible to calculate d-spacing for this sample.

The d-spacing, which represents the periodicity of the lipid bilayer, provided further insights into membrane stability under varying environmental conditions. The bilayer membranes composed solely of diethers exhibited the smallest d-spacing, around 50 Å. At 80% RH, the d-spacing remained relatively stable with increasing temperature, measuring 49.4 Å at 60 °C and 49.8 Å at 80 °C. However, at 95% RH, significant fluctuations were observed, with the d-spacing decreasing from 50.0 Å at 60 °C to 47.0 Å at 80 °C (Figure 4).

In contrast, mixed D/T membranes displayed greater stability, with only minimal fluctuations across conditions. The D/T (1:1) mixture exhibited a d-spacing of 56 Å at 80% RH and 55 Å at 95% RH, maintaining consistency across the tested temperatures (Table S2). Similarly, the D/T (2:1) mixture showed d-spacing values of approximately 55 Å at 80% RH and 54 Å at 95% RH (Figure 4). The D/T (1:1) sample was the only composition tested at 90 °C due to limited beam time for the other samples. Pure tetraether membranes, however, exhibited greater d-spacing variations depending on humidity. At 80% RH, the d-spacing was 51 Å, closely resembling that of diether membranes, while at 95% RH, tetraether membranes expanded significantly, reaching a d-spacing of 56 Å, comparable to that of the D/T mixtures (Figure 4).

### 2.4. NLSD Profiles: Humidity-Induced Structural Modifications

**Figure 5:**
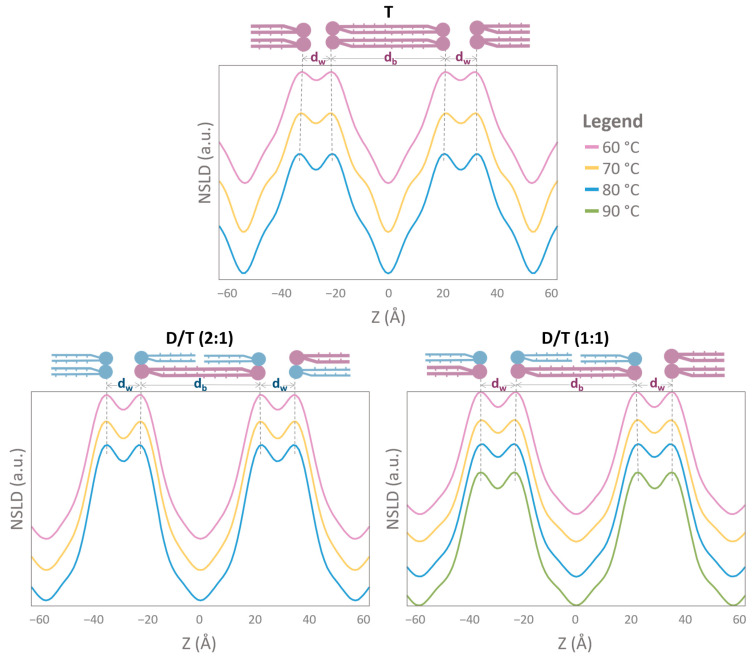
Neutron Scattering Length Density (NSLD) profiles showing two periods of different archaeal membrane compositions. Each plot corresponds to the NSLD profile at one humidity level (80% RH) and different temperatures. The bilayer thickness d_b_ corresponds to the center-to-center distance between headgroups as described in the methods. The water layer thickness d_w_ is calculated according to d = d_w_ − d_b_, d being the d-spacing. The dotted black lines show the shift of the maxima in the NSLD profiles. NSLD profiles were calculated with 4 orders for T and both D/T samples. In these conditions, only 2 orders were detected for the D sample, and the NSLD profile was not calculated. Due to the lack of resolution normally provided by higher diffraction orders, a diether membrane would lead to a wrong or featureless profile. T = tetraether sample, D/T = mixture of diether and tetraether samples at different molar ratios (1:1) or (2:1).

**Figure 6:**
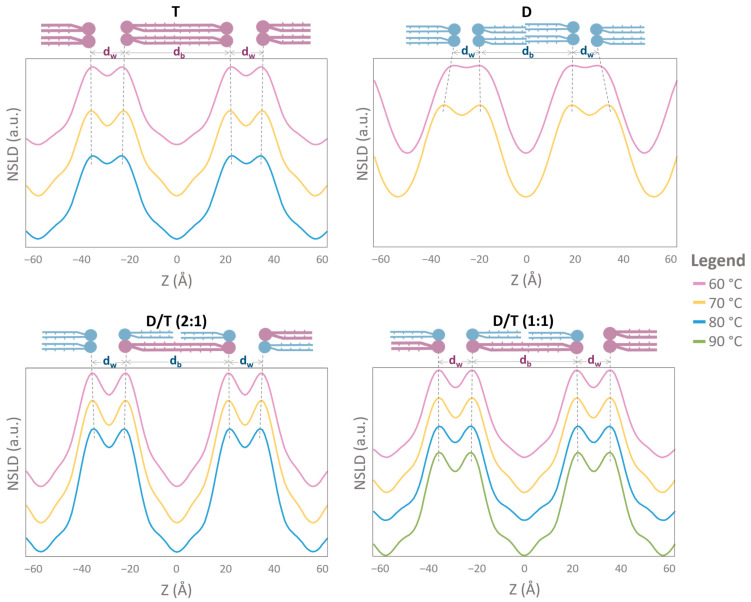
NSLD profiles showing two periods of different archaeal membrane compositions. Each plot corresponds to the NSLD profile at one humidity level (95% RH) and different temperatures. The bilayer thickness d_b_ corresponds to the center-to-center distance between headgroups as described in the methods. The water layer thickness d_w_ is calculated according to d = d_w_ − d_b_, d being the d-spacing. The dotted black lines show the shift of the maxima in the NSLD profiles. NSLD profiles were calculated with 3 orders for D samples and 4 for T, and both D/T samples. A problem occurred during the diether measurement at 80 °C 95% RH. For this reason, it was not possible to calculate NLSD profile for this sample. D = diether sample, T = tetraether sample, D/T = mixture of diether and tetraether sample at different molar ratios (1:1) or (2:1).

To further elucidate membrane structure, NSLD profiles were calculated for each sample under the six tested conditions at 8% D_2_O contrast. The NSLD profiles corresponding to measurements at 80% RH are presented in Figure 5, while those at 95% RH are shown in Figure 6. At 80% RH, only two Bragg peaks were detected for the diether (D) sample, preventing the reliable calculation of its NSLD profile; two diffraction orders do not provide sufficient resolution and could lead to misleading results (Figure 5). At 95% RH, however, three Bragg peaks were observed for the diether sample, allowing the NSLD profiles calculation, except for the measurement at 80 °C, where a sample equilibration issue precluded accurate determination (Figure 6).

**Figure 7:**
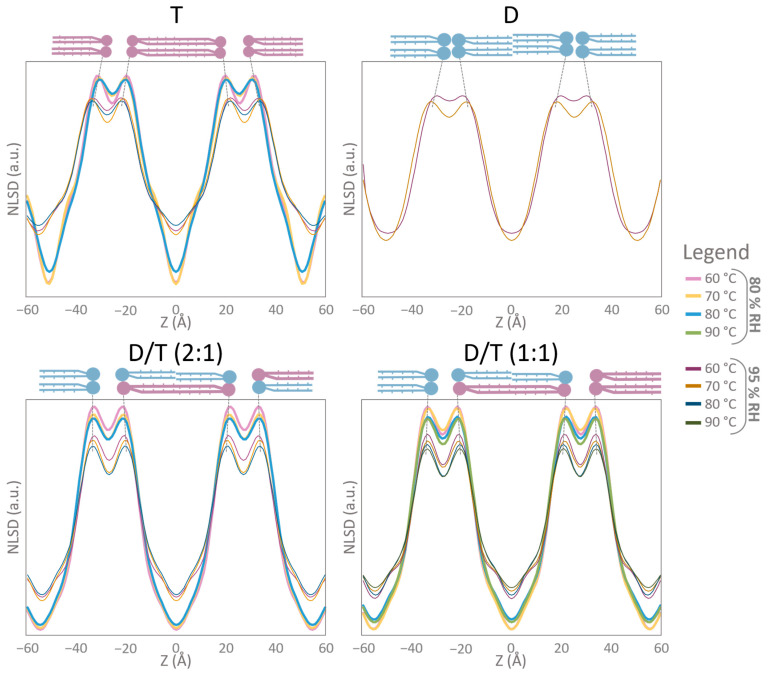
Overlaid NSLD profiles. Each graph corresponds to an NSLD profile at different temperatures and humidities. The dotted black lines show the shift of the maxima in the NSLD profiles. NSLD profiles were calculated with 4 orders for T and both D/T samples. NSLD profiles were calculated with 3 orders for D samples. A problem occurred during the diether measurement at 80 °C 95% RH. For this reason, it was not possible to calculate NLSD profile for this sample. At 80% RH, only 2 orders were detected for the D; the NSLD profile was not calculated. D = diether sample, T = tetraether sample, D/T = mixture of diether and tetraether sample at different molar ratios (1:1) or (2:1).

Across all conditions, NSLD profiles exhibited the characteristic structure of a lipid bilayer, featuring a prominent peak corresponding to the phospholipid headgroup region and a central hydrophobic region (Figure 5 and Figure 6). Notably, a small density “trough” was consistently observed at the bilayer center (Z = 0 Å) in all samples, including tetraether-rich membranes (T and D/T) (Figure 6). Interestingly, NSLD profiles remained relatively unchanged with temperature variations across all tested conditions (Figure 5 and Figure 6). When all NSLD profiles were overlaid, tiny variations were visible (Figure 7). Temperature increases affected the polar headgroup regions (around Z = ±20 Å), resulting in decreased scattering intensity (Figure 7). Humidity had a significant impact on the hydrocarbon chains: in T and D/T membranes, the scattering signal was lower at 95% RH compared to 80% RH, and a distinct “hump” appeared in the hydrocarbon region (around Z = ±10 Å) (Figure 7).

### 2.5. The Specific Contribution of Molecular Groups to NLSD Profiles

**Figure 8:**
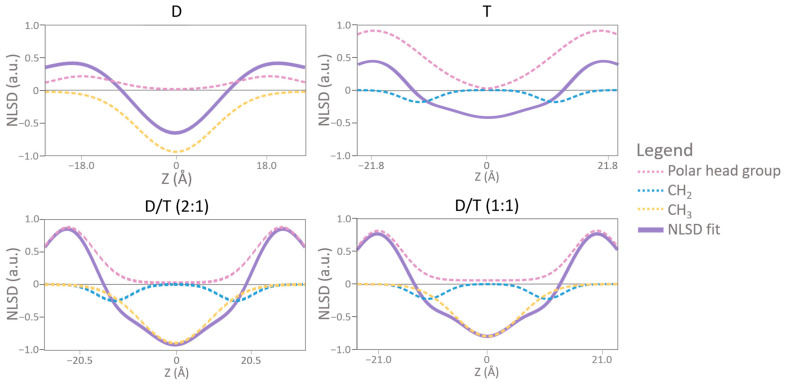
Overlaid of membrane NLSD profile and the specific density profiles of water, headgroups, and methyl groups (CH_2_ and CH_3_) in 95% hydrated membranes. For membranes composed of: D = diether sample, T = tetraether sample, D/T = mixture of diether and tetraether sample at different molar ratios (1:1) or (2:1).

To understand the origin of the observed “trough” and “hump” features, we analyzed NSLD-derived density profiles of hydrocarbon chains (focusing on CH_2_ and CH_3_ groups) and polar headgroups (Figure 8). At 8% D_2_O contrast, the water density profile remained close to zero, allowing us to clearly assess contributions from individual lipid components.

This analysis confirmed that the terminal methyl (CH_3_) groups of diether lipids were responsible for the density “trough” at the membrane midplane (Z = 0 Å). However, in tetraether monolayers, this feature was absent. Instead, CH_2_ groups significantly influenced NSLD profiles at Z = ±10 Å, generating the observed “hump” in tetraether-rich membranes (Figure 8). In D/T mixtures, both CH_2_ and CH_3_ contributions were observed, confirming the expected presence of both diether and tetraether lipids in the membrane. Notably, the trough at Z = 0 Å was more pronounced in D/T (2:1) than in D/T (1:1), reflecting the higher proportion of diethers in this sample. Furthermore, the polar headgroups of tetraether lipids produced a stronger signal (close to 1) compared to those of diethers (approximately 0.2), suggesting that tetraether headgroups contribute more substantially to the overall membrane density (Figure 8).

### 2.6. Membrane Thickness and Water Layer Characteristics

**Figure 9:**
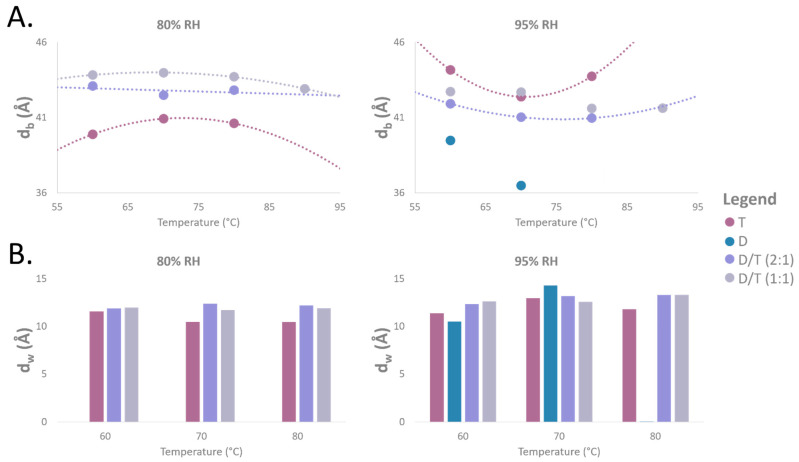
Thickness measurements for the different samples. (**A**) Membrane thickness (d_b_) as a function of temperature for different lipids ratios, measured at 80% and 95% RH. Error for d_b_ measurements is ±0.5 Å. (**B**) Water thickness (d_w_) as a function of temperature for different lipids ratios, measured at 80% and 95% RH. Error for d_w_ measurements is ±0.5 Å. D = diether sample, T = tetraether sample, D/T = mixture of diether and tetraether sample at different molar ratios (1:1) or (2:1). No data was available for the diether sample at 80% RH for all tested temperatures and at 80 °C with 95% RH.

From NSLD profiles, we extracted key membrane parameters, including bilayer thickness (d-spacing), Gibbs–Luzzati bilayer thickness (d_b_), and water layer thickness (d_w_) (Figure 9A,B). The measured thickness of the diether membranes (determined only at 95% RH) decreased slightly with increasing temperature, from 39 Å at 60 °C to 36 Å at 70 °C, while the water layer thickness increased from 10 Å at 60 °C to 14 Å at 70 °C (Figure 9A,B).

For the pure tetraether sample at 80% RH, the membrane thickness was similar to that of diether membranes, ranging from 40 Å at 60 °C to 41 Å at 80 °C. However, the water layer thickness decreased from 12 Å at 60 °C to 10 Å at 80 °C. In contrast, at 95% RH, both the membrane and water thickness increased, reaching 44 Å and 11–12 Å, respectively (Figure 9A,B). Meanwhile, mixed D/T membranes maintained highly stable d_b_ and d_w_ values, regardless of temperature or humidity fluctuations. For example, the D/T (2:1) mixture exhibited a membrane thickness that varied slightly from 43 Å at 60 °C to 42 Å at 70 °C, while the D/T (1:1) mixture remained stable at 44 Å. The water layer thickness in both D/T samples was consistently measured around 12 Å across all conditions (Figure 9A,B).

## 3. Discussion

### 3.1. On the Importance of Natural Polar Headgroups on Membrane Stability

Several of the previous studies conducted to measure the behavior of the archaeal bilayer membranes have been performed with synthetic archaeal-like lipids bearing headgroups such as phosphatidylethanolamine (PE) or phosphatidylcholine (PC) [31,45,46]. While providing invaluable information on membrane stability as a function of temperature or high hydrostatic pressure, the lipids used harbor polar headgroups that are not the most relevant for archaeal membrane studies. Indeed, PC and PE headgroups are common in Bacteria and Eukarya but are rarely found in Archaea. PE is predominant in *E. coli* membranes [47], but in Archaea, it has only been identified in a few species, including *Methanosarcina barkeri*, *Archaeoglobus fulgidus*, and *Thermococcus kodakarensis* [48,49,50]. PC, common in eukaryotic cells, is even rarer in archaeal membranes, with reports limited to *Methanopyrus kandleri* [51]. Instead, archaeal membranes predominantly feature phosphatidylinositol (PI), phosphatidylglycerol (PG), or more complex structures such as oligosides, sulfur-substituted sugars, or methylphosphate derivatives of PG. However, due to their complex synthesis and lack of commercial availability, synthetic analogs of these lipids remain scarce. Several studies have shown that polar headgroup size and structure critically influence membrane packing and stability. For instance, the smaller PE headgroup results in tighter membrane packing, whereas the larger PC headgroup produces a less densely packed membrane [52,53]. Given the significant impact of headgroup structure on membrane behavior, the current experiment with purified natural archaeal lipids was conducted to better mimic biological reality and evaluate the impact of representative polar headgroup on membrane stability as a function of temperature stress. The purified natural diether (*P. furiosus* PHex-DGD) and tetraether (*S. acidocaldarius* P-Hex_2_-GDGT/P-Hex_2_-Calditol-GDGT) lipids (Figure 1 and Figure 2) harbored essentially glucose polar headgroups (89% in diethers and 98% in tetraethers).

The ability of our natural lipids to form oriented bilayer structures was confirmed using neutron diffraction on the D16 instrument at the ILL. Strong diffraction signals were obtained across all tested conditions, even at elevated temperatures of 80 °C and 90 °C, which was the upper limit of the BerILL setup (Figure 3 and Figure S1). This level of stability contrasts with previous findings on synthetic archaeal-like diethers, such as DoPhPC and DoPhPE, which exhibited poorly structured bilayers at comparable temperatures [31,45,46]. For instance, a 9:1 DoPhPC:DoPhPE mixture maintained organized membrane structures only up to 70 °C, while archaeol-based lipids with PI headgroups lost lamellar organization at temperatures above 55 °C [54]. In contrast, our PHex-DGD diether sample exhibited superior stability, producing three distinct Bragg peaks at 60 °C and 70 °C at 95% RH (Figure 3). Because DoPhPC, DoPhPE, DoPhPI, and PHex-DGD share the same diether hydrophobic core, the key structural difference among them lies in their polar headgroups. This suggests that archaeal membrane stability is strongly influenced by headgroup chemistry, which is congruent with previous observations on bacterial membranes [53]. One plausible explanation for the superior stability of our natural lipids is the ability of PHex headgroups to engage in extensive hydrogen bonding with water molecules, thereby stabilizing the lamellar phase through inter- and intramembrane interactions, compared to PE or PC head groups. Even subtle variations in hexose composition, such as differences between glucose and inositol, have been shown to impact bilayer organization. Despite their structural similarity, our results suggest that P-Glucose confers greater membrane stability than PI. The precise reason for this difference remains unclear, but it may stem from variations in their hydrogen bonding patterns or steric effects that influence membrane packing.

These findings further highlight the necessity of using natural lipids rather than synthetic analogs when exploring archaeal membrane biophysics. Our results demonstrate that the enhanced stability of our reconstructed membranes is closely linked to the headgroup composition of the natural lipids used. This is consistent with previous observations on archaeal lipid mixtures. For instance, in the acidophile *Picrophilus oshimae*, intact polar lipids extracted from the organism were unable to self-organize into liposomes at pH values above 4 but formed highly stable, impermeable membranes at lower pH [55]. Similarly, purified lipids from extreme halophiles like *Halobacterium halobium* failed to form liposomes in the absence of NaCl, while at high salt concentrations, liposomes exhibited remarkable membrane stability. This was attributed to the presence of the halophile-specific polar headgroup, phosphatidylglycerophosphate methyl ester (PGP-Me), which prevents bilayer aggregation through steric repulsion [56]. These findings highlight how the polar headgroups of natural lipids have evolved to counteract the environmental conditions in extremophilic Archaea.

Our study provides compelling evidence that phosphoglucose headgroups, in particular, play a crucial role in the thermal stability of hyperthermophilic membranes. While previous research has predominantly focused on modifications of the hydrophobic core, such as increased tetraether content or cyclopentane ring incorporation, to enhance membrane rigidity at high temperatures [21,23], our results highlight the significant role of the headgroup in these adaptation mechanisms. It is important to recognize that native archaeal membranes are composed of a complex array of lipids with diverse polar headgroups, making it challenging to extrapolate general principles from single-lipid systems. Nonetheless, our findings underscore the need to consider the interplay between headgroup composition and hydrophobic core modifications when studying archaeal membrane stability. Future investigations integrating molecular dynamics simulations and biophysical assays on mixed-lipid systems will be critical for further unraveling the contribution of lipid diversity to archaeal membrane adaptation. It is interesting to note that the diversity of polar headgroups in the model archaeal species *Pyrococcus furiosus* is yet largely unknown. It will, therefore, be important to develop methods to access the diversity of membrane lipids in this strain or be able to assay the importance of these novel lipids on membrane properties.

### 3.2. Impact of the Diether to Tetraether Ratio on Membrane Thickness

Tetraether membranes exhibit unique structural characteristics that distinguish them from traditional bilayer membranes. Unlike diether membranes, which consist of two separate lipid leaflets, tetraether lipids span the entire membrane, forming a monolayer structure. Due to this structural feature, monolayer membranes composed of tetraether lipids were expected to be thinner than bilayer membranes, which contain a slip plane between their lipid layers. However, our findings challenge this assumption. Pure tetraether sample exhibited d-spacing of 55.3 Å at 95% RH at 70 °C (Figure 4), which is consistent with earlier findings from the Winter and Chong teams, who reported d-spacing values of 56 Å at 74 °C for *Sulfolobus acidocaldarius* polar lipid fractions using Small-Angle X-ray Scattering (SAXS) and High-Pressure FT-IR Spectroscopy [57]. Interestingly, at lower humidity (80% RH), the d-spacing of tetraether membrane decreased to 51.5 Å at 70 °C, which is much closer to the smaller d-spacing observed for diether membranes (ca. 50 Å) (Figure 4). This result was unexpected, as the slip plane between diether lipid layers was anticipated to result in thicker membranes compared to the tetraether monolayers. Membrane thickness measurements provided further insight: diether membranes exhibited a thickness of 36 Å at 70 °C, whereas tetraether membranes measured 42 Å (Figure 9). These results align with molecular dynamics (MD) simulations predicting a thickness of 38 Å for diether diphytanyl phosphatidylcholine membranes and 40 Å for acyclic tetraether phosphatidylcholine membranes at 25 °C [58].

In contrast to initial expectations, diether membranes appear thinner than tetraether membranes. This slightly greater thickness in tetraether membranes may be attributed to the larger polar headgroups compared to diethers (Figure 1 and Figure 2). Since thickness measurements reflect head-to-head distances, variations in membrane thickness may primarily arise from differences in headgroup composition rather than the hydrophobic core. Indeed, NSLD-derived density profiles demonstrated that tetraether polar headgroups produced a significantly stronger signal (~1) compared to diether headgroups (~0.2), further supporting their role in overall membrane density (Figure 8). Notably, diether membranes were predominantly composed of P-Glucose headgroups, whereas tetraether lipids carried larger moieties, primarily composed of Calditol and P-Glucose-Glucose headgroups (Figure 1 and Figure 2). These differences suggest that membrane thickness and stability cannot be solely attributed to the monolayer vs. bilayer nature of the lipids but are also influenced by headgroup size and composition.

### 3.3. Impact of the Diether to Tetraether Ratio on Membrane Stability

A particularly intriguing finding was the presence of a small “trough” at the membrane center (Z = 0 Å) in NSLD profiles for all samples, including those rich in tetraether lipids (T and D/T) as well as the diether sample (Figure 7). This feature, typically attributed to the density of hydrogen atoms at terminal methyl groups, is commonly associated with the slip plane in bilayer membranes [59,60]. While this trough was expected for the diether membrane, its presence in tetraether-rich membranes was surprising, as tetraether lipids span the entire membrane and lack a classical bilayer midplane interface.

We hypothesized that this “trough” resulted from the shorter distance between the two methyl groups at the center of the hydrophobic chains due to the head-to-head condensation of two diether molecules to generate the tetraether lipid. The distance is one carbon shorter, potentially creating a localized electron density defect responsible for the observed trough [61]. Interestingly, this interpretation differs from electron density (ED) profiles obtained from MD simulations of tetraether membranes, which do not exhibit such a feature [62]. The discrepancy likely arises from fundamental differences in how neutrons and electrons interact with molecular structures. Furthermore, the presence of a “trough” in NSLD profiles of both diether and tetraether membranes can also suggest that the leaflet interface in diether membranes may be smaller than previously described (Figure 7). The closer proximity of terminal methyl groups in diether membranes could enable interactions such as hydrogen bonding or Van der Waals attractions, which could contribute to increased membrane stability. Density profile analysis confirmed that the terminal methyl (CH_3_) groups of diether lipids are responsible for the “trough” at the bilayer midplane, while in tetraether membranes, this contribution is absent (Figure 8).

Moreover, at 95% RH, NSLD profiles revealed a more pronounced “hump” in the hydrocarbon chain region for the tetraethers-containing membranes compared to those at 80% RH (Figure 7). This hump, visible at Z = ±10 Å, suggests branched chains reorganization in the lipid midplane and changes in hydrocarbon conformation (Figure 7). This region is also associated with higher SLD carbonyl and phosphate groups [62,63]. This modification is more noticeable in the pure tetraether sample, meaning that the hydrocarbon chain is undergoing conformational changes while the humidity increases. Further analysis of density profiles confirmed that CH_2_ groups significantly influenced NSLD profiles at Z = ±10 Å, contributing to the “hump” observed in tetraether-rich membranes (Figure 8). These findings suggest that tetraether membranes undergo structural reorganization in response to humidity variations, further supporting their crucial role in maintaining membrane stability under high humidity conditions.

### 3.4. Impact of the Cyclopentane Rings on Membrane Properties

Cyclopentane rings in tetraether lipids play a critical role in modulating membrane properties, particularly membrane thickness and rigidity. MD simulations demonstrated that membrane thickness increases with ring number: ringless membranes measured 40 Å, while membranes with four rings reached 42.6 Å [58]. Additionally, calditol-GDGT membranes exhibited a decrease in thickness from 62.7 Å (ringless) to 45.8 Å (eight rings) [16,64]. The tetraether lipid mixture used in this study contained a diverse population of cyclized tetraethers, with varying ring numbers from 0 to 6. While it was not possible to distinguish the contributions of individual ring configurations, our core lipid characterization confirmed that most molecules contained between two and five cyclopentane rings (Figure 2C). The membrane thickness measured in this study (44 Å at 95% RH and 60 °C, Figure 9) closely aligns with values reported for membranes composed of cyclized tetraether lipids [58].

While previous studies have shown that the proportion of cyclized tetraethers increases in hyperthermophilic archaeal membranes to enhance rigidity [65,66], our study demonstrates that monolayer membranes composed of cyclized tetraethers exhibit exceptional thermal stability. These findings highlight the unique structural adaptability of tetraether-based membranes. Due to their spanning monolayer structure and the presence of cyclopentane rings, tetraether lipids display remarkable resilience under extreme heat stress, maintaining a stable membrane thickness of 44 Å at 95% RH across temperatures ranging from 60 °C to 80 °C (Figure 9).

### 3.5. Diether and Tetraether Lipids Form True Mixtures in the Reconstructed Membranes

One of the key questions regarding membranes composed of a mixture of bilayer- and monolayer-forming lipids is how these components organize spatially and interact to form a functional membrane. Interestingly, our results indicate that diether–tetraether (D/T) mixtures exhibited a stable d-spacing around 55 Å, closely resembling the values observed for pure tetraether membranes at 95% RH, while diether membrane d-spacing remained stable at 49 Å (Figure 4). These findings were counterintuitive, as the difference in molecular length between one tetraether molecule and two diether molecules should have resulted in an intermediate d-spacing. Instead, the results suggest that diethers and tetraethers interact cooperatively, forming an organized and stable membrane structure that more closely resembles pure tetraether membranes. Interestingly, the D/T (1:1) mixture exhibited a larger d-spacing (55.3 Å) than the D/T (2:1) mixture (54.3 Å) under the same conditions (Figure 4). This suggests that increasing the proportion of diethers in the membrane leads to a decrease in d-spacing, highlighting how the lipid ratio influences membrane architecture.

Additionally, NSLD profiles confirmed that both D/T mixtures displayed the characteristic bilayer organization, with a prominent peak corresponding to the phospholipid headgroup region and a central hydrophobic region. Similar to pure tetraether membranes, NSLD profiles for the mixtures exhibited a small “trough” at the bilayer center (Z = 0 Å), as well as a pronounced “hump” in the hydrocarbon chain region when humidity increased (Figure 7). This hump, observed within a region of ±10 Å from the bilayer midplane, suggests structural adjustments within the lipid core. NSLD-derived density profiles of hydrocarbon chains further confirmed that both CH_2_ and CH_3_ groups contributed to the NSLD profiles of D/T membranes, confirming the presence of both lipid types in the membrane. Notably, the trough at Z = 0 Å was more pronounced in the D/T (2:1) mixture than in the D/T (1:1) sample, reflecting the higher proportion of diethers in this composition (Figure 8).

### 3.6. Lipid Diversity as a Key to Membrane Stability and Adaptability

Having confirmed that D/T samples represent true mixtures of diether and tetraether lipids, we further investigated their membrane characteristics. Ω-scans and 1D neutron diffractograms revealed that D/T mixtures consistently exhibited four well-defined Bragg peaks, indicative of a well-organized structure, in contrast to the broader peaks observed for pure diether and tetraether samples (Figure 3A,B). This suggests that mixed lipid membranes are better equipped to adapt to environmental stresses. Moreover, D/T mixtures maintained stable d-spacing values across tested temperatures and humidity conditions, remaining around 55 Å. In contrast, pure tetraether membranes showed greater variability, with d-spacing values ranging from 51 Å at 80% RH to 55 Å at 95% RH, while diether membrane d-spacing remained stable at 49 Å under both humidity conditions (Figure 4).

Stacked NSLD profiles revealed variations in the polar headgroup region (Z = ±20 Å) (Figure 7). Increasing temperature led to a decrease in scattering signal, which was particularly evident in both D/T mixtures (Figure 7). Higher temperatures resulted in membrane disorder and reduced Bragg peak intensity. As this membrane region has been associated with inter-lamellar interactions, it appears that temperature fluctuations cause significant reorganization of polar headgroups, particularly in mixed membranes compared to pure tetraether samples (Figure 7). Hydration also affected all samples, with neutron scattering signals appearing less intense at 95% RH compared to 80% RH (Figure 7). However, membrane thickness measurements further underscored the stabilizing effect of lipid diversity (Figure 9). The D/T (2:1) mixture exhibited a stable bilayer thickness of 42 Å at 60 °C and 41 Å at 80 °C at 95% RH, while the D/T (1:1) mixture remained at 43 Å at 60 °C and 42 Å at 90 °C (Figure 9). In comparison, diether membrane thickness decreased from 39 Å at 60 °C to 36 Å at 70 °C, whereas tetraether membrane thickness remained stable at 44 Å at both 60 °C and 80 °C. Water layer thickness was similarly stable in D/T samples, maintaining values of 12–13 Å at 95% RH (Figure 9). In contrast, water layer thickness fluctuated for pure lipid membranes, varying from 10 Å at 60 °C to 14 Å at 70 °C for diethers and from 11 Å at 60 °C to 12 Å at 70 °C for tetraethers (Figure 9). These observations indicate that incorporating diethers into monolayer-forming membranes or tetraethers into bilayer-forming membranes stabilizes key membrane parameters compared to pure lipid membranes (Figure 9A,B). The combination of rigid and flexible lipid components in mixed membranes appears to optimize stability and adaptability, which is likely crucial for survival in highly fluctuating environments. Moreover, as expected, monolayer membranes displayed greater structural stability than bilayer membranes at high temperatures. This was evidenced by the higher degree of membrane organization in tetraether samples, which exhibited four Bragg peaks, compared to diether membranes, which displayed a maximum of three Bragg peaks, indicating a lower level of structural order.

These findings shed new light on the widely recognized homeoviscous adaptation observed in hyperthermophilic Thermococcales Archaea, where increasing tetraether content and reducing diethers enhances membrane rigidity and thermal stability [21,23,67,68]. In these species, which thrive at temperatures close to 100 °C, the presence of diether lipids may play an unexpected but crucial role in enabling adaptation to highly fluctuating thermal conditions. Given that these organisms inhabit environments ranging from hydrothermal fluids exceeding 350 °C to deep-sea waters at ca. 2 °C, membrane plasticity and stability must be tightly regulated and necessitate a sophisticated membrane homeoviscous adaptation mechanism. This study provides the first experimental insights into the structural organization of archaeal tetraether membranes composed of naturally purified lipids from *Sulfolobus acidocaldarius*. Furthermore, we characterized the effect of diether lipid incorporation into monolayer membranes. If the current structural analysis was exclusively based on neutron scattering diffraction data, this technique was particularly advantageous, as it is non-destructive and requires minimal lipid sample quantities, critical given the limited availability of purified lipids (9 mg of tetraethers and 12 mg of diethers). However, this approach also imposed certain limitations. For example, the BerILL setup did not allow for testing temperatures above 90 °C or humidity levels beyond 95% RH. Despite these constraints, our data align with trends previously reported using MD simulations, SAXS, and high-pressure FT-IR spectroscopy. Future studies should integrate complementary techniques to refine our understanding of archaeal membrane monolayer structure and the effects of diether incorporation. Dynamic light scattering, which enables the determination of diffusion coefficient and hydrodynamic radius of vesicles in solution, as well as solid-state nuclear magnetic resonance (NMR) and SAXS experiments, would be valuable for further elucidating the structure and dynamics of archaeal lipid membranes.

## 4. Materials and Methods

### 4.1. Experimental Design

To assay the relative contribution of diether and tetraether on membrane features and properties, we have performed our measurements on natural, isolated polar lipids rather than on lipid mixtures, synthetic lipids, or homologs. We synthesized 4 types of samples with various diether-to-tetraether (D/T) ratios. In addition to the purified diether (6 mg) and tetraether (3 mg), which served as pure pole controls, 2 D/T mixtures were prepared: a 2:1 (molar) D/T mixture containing 3 mg of diether and 3 mg of tetraether, and a 1:1 (molar) D/T mixture containing 1.5 mg of diether and 3 mg of tetraether. We performed neutron diffraction experiments. At least 3 temperatures (60 °C, 70 °C, and 80 °C), two relative humidities (80% and 95% RH), and two D_2_O contrasts (100% and 8%) were tested (Table S1).

### 4.2. Archaeal Lipids

Diether lipids were purified from the hyperthermophilic archaeon *Pyrococcus furiosus* strain DSM3638 (Deutsche Sammlung von Mikroorganismen und Zellkulturen, DSMZ) as described in the Supplementary Materials (Figure S3), while tetraether lipids were purified from the acidophilic and thermophilic archaeon *Sulfolobus acidocaldarius* strain MW001 [69] (Figure S4).

### 4.3. Archaeal Membrane Reconstruction

Samples with various diether-to-tetraether (D/T) ratios were studied as a multi-stack of lipid bilayers on one-sided polished ultraclean silicon wafers with a thickness of 275 ± 25 μm purchased from Si-Mat (Kaufering, Germany). The wafers were previously cut to produce a rectangular shape (4 cm × 2.5 cm) to fit the sample compartment. Silicon wafers were rinsed with TCM and dried by a N_2_. Lipid samples were spread on a silicon wafer and dried overnight under a vacuum.

Samples were transferred to the high-precision BerILL humidity chamber [70] and mounted vertically on a manual 4-axis goniometer head (Huber, Berching, Germany) set in the humidity chamber and pre-aligned using a laser-based optical setup. Each sample was incubated in the humidity chamber for at least 3 h prior to the first data collection. Among the different conditions, 1 h of equilibrium was respected to ensure a constant d-spacing and constant intensity of Bragg reflections during the experiment.

### 4.4. d-Spacing Calculation

Neutron diffraction data were collected at the recently upgraded D16 cold neutron diffractometer of the Institut Laue-Langevin (ILL, Grenoble, France) [71]. The instrument design has been revisited, with a new secondary spectrometer to allow the installation of a new curved wide-angle 2D detector based on the trench technology developed at ILL [72]. The new detector has a pixel resolution of 1.5 × 2.0 mm^2^ (hor. × vert.), providing an angular resolution of 0.075 × 0.1 deg. 2θ resolution at a distance of 1150 mm.

To collect the data, the vertical sample plane is illuminated by the horizontally collimated and vertically focused incident beam with an adjustable angle of incidence set by the sample angle Ω. The beam is scattered in different directions at angles 2θ from the incident beam. For each Ω, the 2θ dependent intensity is collected. By rotating the sample stage, Ω-scans (rocking curves) were performed in steps of 0.05 deg. from −1 to 13 deg. The intensity was corrected for the detector pixel efficiency resulting from a water calibration run. For each Ω-scan, 2D images obtained at a given Ω step were reduced to 1D by vertical integration of the intensity in an ROI (2θ_y_ vs. 2θ_x_ range) centered vertically on Bragg reflections and stacked to produce the reciprocal space map. These steps, from the Ω-scans’ raw data to the production of the 2D reciprocal space maps, were performed using the Mantid software (v6.11.0), Daily version [73]. The analysis of the reciprocal space maps was performed in Igor Pro 8.0 (WaveMetrics, Lake Oswego, OR, USA) using a dedicated procedure written by T. Hauss for the new D16 detector and data format.

The analysis consisted of the integration of the intensity along the specular direction to produce the I vs. 2θ curve. Bragg peaks were then fitted using the Multipeak fitting tool of Igor Pro 8.0, the positions and intensities of which were used for the determination of the period of the lamellar membrane stack (d-spacing) and the reconstruction of the membrane density profile in real space. Here, q_z_ is the scattering vector normal to the bilayer planes related to the scattering angle (2θ) defined as

q_z_ = 4πsin(θ)/l, (1)

When several orders of diffraction were observed (up to 4), a linear fit of the peak positions vs. Bragg order (n) was performed. The slope of which was used to determine the d-spacing (d) using the Equation (1), where λ is the incident neutron beam wavelength (4.487 Å): d = λ/Δq. When only a single Bragg reflection was observed, the d-spacing was calculated using the first-order peak according to d = 2π/q_z_.

### 4.5. Neutron Scattering Length Density (NLSD) Profiles

NSLD profiles were calculated according to [74] from the integrated intensities of Bragg peaks corrected for the neutron absorption (C_abs_), the Lorentz correction (C_Lor_), and the neutron flux correction (C_flux_), resulting in the corrected discrete structure factor of order n:

(2) $$F_{n}=\sqrt{I_{h}C_{\mathrm{abs}}C_{\mathrm{flux}}C_{\mathrm{Lor}}},$$

where I_h_ is the intensity of the Bragg peak at the order h. The corrections are given by

(3) $$C_{\mathrm{abs}}=\frac{\alpha }{1-e-\alpha },\alpha =\frac{2µt}{sin\theta },$$

(4) $$C_{\mathrm{Lor}}=sin(2\theta ),$$

(5) $$C_{\mathrm{flux}}=\frac{1}{erf}(\frac{Lsin\left(\theta \right)}{\sqrt{8}\delta }),$$

where *t* is the sample thickness, calculated from the deposited amount of dry lipid and the sample area, and 2δ is the beam width [75].

Finally, the NSLD was calculated using

(6) $$\rho \left(z\right)=\frac{2}{d}\sum_{h=1}^{h=n}V_{h}F_{h}\cos\left(\frac{2\pi hz}{d}\right),$$

where *z* is the specular direction perpendicular to the bilayer planes, *h* is the diffraction order, *n* is the highest order detected, and V*_h_* corresponds to the phase of the structure factor *h*. Using the 8% D_2_O contrast, lipid headgroups are highlighted due to the zero NSLD of water and the negative NSLD of aliphatic chains.

We tested the different hypotheses for the discrete structure factor signs to obtain an NSLD profile with a minimum at *z* = 0 and a maximum NSLD for the polar head regions. The best agreement with these constraints was [−, −, +, −] for V_h_. From these profiles, the Gibbs–Luzzati bilayer thickness (d_b_) can be directly extracted [76] as the center-to-center distance between polar headgroup layers [45]. The thickness of the water layer between the lipid bilayer (d_w_) is calculated from the known d-spacing and the bilayer thickness according to d_w_ = d − d_b_, as illustrated (Figure S2).

### 4.6. Statistics

Neutron scattering is an inherently statistical technique with errors based on Poisson statistics. The error associated with the d-spacing was calculated by propagating the uncertainty from the slope of the linear fit to the data. Raw data are available at https://doi.org/10.5291/ILL-DATA.8-02-991 and https://doi.org/10.5291/ILL-DATA.8-02-1046 following ILL’s data management policy.

## 5. Conclusions

Our study highlights the critical role of phospholipid polar headgroups in membrane stability, particularly under extreme environmental conditions. We provide compelling evidence that phosphoglucose headgroups, in particular, are essential for the thermal stability of hyperthermophilic membranes. While previous research has largely focused on modifications to the hydrophobic core, our findings emphasize the significant contribution of headgroup composition to membrane adaptation. This suggests that a full understanding of archaeal membrane stability must consider natural lipid diversity, as these lipids exhibit remarkable resilience under extreme conditions.

Furthermore, our results demonstrate the essential role of lipid mixtures in optimizing membrane stability and adaptability. We show that membranes composed of mixed diethers and tetraethers are more structurally organized and stable than those formed by pure lipid systems. This stability is maintained across varying temperatures and humidity levels, highlighting further the advantage of lipid diversity in hyperthermophilic Archaea. While most hyperthermophiles naturally produce a mix of diethers and tetraethers, some species rely on a single lipid type. Our findings suggest that single-lipid membranes, though viable at high temperatures, lack the robustness of mixed-lipid systems, indicating that these species may rely on additional membrane components to enhance membrane stability. For example, in Thermococcales, this is the role played by apolar polyisoprenoid lipids to stabilize bilayer membranes [23].

Interestingly, our study shows that diethers play a crucial role in the stability of archaeal monolayer membranes. Incorporating diethers into monolayer-forming membranes or tetraethers into bilayer-forming membranes improves key membrane properties compared to pure lipid systems. The combination of rigid and flexible lipid components in mixed membranes appears to be a key strategy for optimizing both stability and adaptability essential for survival in dynamic environments. Beyond deepening our understanding of archaeal membrane adaptation, these findings may have significant implications for bioinspired synthetic membranes. The exceptional stability of mixed lipid systems offers exciting possibilities for developing heat-resistant biomaterials with applications in biotechnology, medicine, and industry. The use of archaeal tetraether lipid liposomes (archaeosomes) as drug delivery systems has, for example, been proposed. Introducing a fraction of archaeal diether lipids may help increase their stability, a key property for certain treatments such as in hyperthermia-induced cancer therapies [77]. By enhancing membrane stability, the D/T lipid mixture could improve the efficiency and longevity of gene delivery systems, making them promising candidates as next-generation gene transport vehicles. Additionally, the thermal stability of ether lipids from extremophilic Archaea makes them ideal for high-performance lubricants that require neither preheating nor carrier lubricants, offering significant advantages for industrial machinery operating under extreme conditions [78,79].

Looking ahead, future research integrating X-ray scattering, molecular dynamics simulations, and computational modeling will provide deeper insights into how lipid composition influences membrane resilience. Additionally, exploring how mixed D/T membranes respond to multiple stressors, such as salinity and pH, is crucial, as many Archaea are multi-extremophiles. It will also be essential to measure the relative contribution of the different polar headgroups of archaeal lipids since it may provide lipids with novel features for biotechnology. These advances will pave the way for innovative applications where bioinspired lipid architectures can enhance membrane stability across a wide range of biotechnological, medical, and industrial processes.

## Data Availability

All neutron data are publicly available on ILL servers and are accessible with the following DOIs: https://doi.org/10.5291/ILL-DATA.8-02-991 and https://doi.org/10.5291/ILL-DATA.8-02-1046.

## Supplementary Materials

The following supporting information can be downloaded at https://www.mdpi.com/article/10.3390/ijms26073045/s1. References [43,80,81,82] are cited in the Supplementary Materials.
