# Supplementary material for: Bilayer-Forming Lipids Enhance Archaeal Monolayer Membrane Stability
Source: Int J Mol Sci. 2025 Mar 26;26(7):3045. doi: 10.3390/ijms26073045 (PMC11988840; doi:10.3390/ijms26073045)
Supplement: Supplementary file 1 [file ijms-26-03045-s001.zip › ijms-3512897-supplementary.pdf]

Supplementary Materials

Bilayer-Forming Lipids Enhance Archaeal Monolayer Membrane Stability

Margot Saracco [1], Philippe Schaeffer [2], Maxime Tourte [3], Sonja-Verena Albers [3], Yoann Louis [1], Judith Peters [4,5,6], Bruno Demé [4], Stephane Fontanay [1] and Philippe M. Oger [1,*]

S1. Materials and Methods

S1.1. Diether Lipid Purification

P. furiosus cultures were carried as described previously [80] in 18 L of Defined Cellobiose (DC) minimal medium at 98 °C in a 20 L fermenter under strict anaerobiosis. The culture was inoculated to 1.106 cells/mL and incubated at 98 °C for 20 h. Cells were recovered by centrifugation in 1 L batches, rinsed twice in sterile 3 % NaCl aqueous solution, and lyophilized overnight.

Dried cell pellets were extracted with 40 mL of a monophasic mixture of methanol (MeOH)/trichloromethane (TCM)/purified water (1:2.6:0.16; v/v/v) using a sonication probe for 10 min. After centrifugation (4000x rpm, 5 min), the supernatant was collected, and the extraction procedure was repeated twice. The supernatants were pooled and the solvents were removed under reduced pressure using a rotary evaporator. Finally, the solvent extract was solubilized using a mixture of MeOH/TCM (1:1; v/v), transferred into a 2 ml vial, and the solvents removed under an N2 stream. Extracted intact polar lipids (IPL) were kept at -20 °C until lipid purification.

A two-dimensional thin-layer chromatography purification step of the IPL extract from P. furiosus was performed using silica gel pre-coated glass-backed plates (60 Å silica-gel, 20 cm x 20 cm plates). Polar lipids containing IPL from *P. furiosus* were separated by first developing the plate using the 1D mobile phase mixture TCM/MeOH/water (75:25:2.5; v/v/v) for 45 min in the first direction. After allowing sufficient time for drying, the plate was developed, at right angles to the first development using the 2D mobile phase TCM/MeOH/acetic acid/water (80:9:12:2; v/v/v/v) for 1.5 h [52]. All components were detected using iodine and collected by scrapping the silica gel zone having the desired retention factor (Rf) zone. Purified lipids were extracted from the silica gel with a mixture of TCM/MeOH (1:1; v/v) (Figure S3).

The composition of each Rf zone recovered was verified by HPLC-ESI-MS using an Inertsil Diol column (2.1 mm x 250 mm; 5 µm; GL Sciences) equipped with a pre-column with the same stationary phase. The separation was carried out in solvent gradient mode including a mobile phase solvent A (isopropanol -IPA-/water/formic acid/aqueous ammonia 88:10:0.12:0.04; v/v/v/v) and solvent B (n-heptane -n-C7-/IPA/formic acid/aqueous ammonia (79:20:0.12:0.04; v/v/v/v). An Agilent 1100 binary HPLC pump equipped with an autosampler and a

thermostated oven set at 30 °C was used. Chemstation software (version Rev. 3.01.01.SR1) was used to control the HPLC analyses. IPL were eluted at a constant flow rate of 0.2 mL/min from 100% solvent B to 66% solvent B in 18 min, then maintained for 12 min, followed by 35% solvent B in 15 min, maintained for 15 min, and finally back to 100% solvent B in 2 min, followed by a 20 min stabilization period of the column (modified after [81]). An Esquire 3000Plus or an HCT ion trap mass spectrometer (Bruker) equipped with an electrospray ionization (ESI) source used in positive and negative modes were used. The conditions for the MS analyses were as follows: nebulizer pressure 30 psi, cone tension 40 V, drying gas (N2) flow 8 L/min and temperature 340 °C, capillary voltage 5 kV (negative mode) and -4 kV (positive mode), mass range m/z 500–2000 (Esquire 3000Plus) or m/z 500-3000 (HCT). Mass spectra (protonated -positive mode- and deprotonated -negative mode-) molecular ion masses of IPL were analyzed using Bruker Data Analysis software (version 4.2).

S1.2. Tetraether Lipid Purification

Sulfolobus acidocaldarius cells were grown aerobically at 75 °C with shaking in Brock's minimal medium in the presence of 20 $\mu g.mL^{-1}$ uracil (Sigma-Aldrich) [82]. Cells were recovered by cross-flow filtration, rinsed twice, and lyophilized overnight.

Sulfolobus acidocaldarius cell pellets were provided by M. Tourte and S.V. Albers from the Molecular Biology of Archaea team (Institute of Biology II, University of Freiburg, Germany). Most previous studies on tetraether lipids have been performed on the polar lipid fraction E (PLFE) of *S. acidocaldarius*. Our preliminary HPLC-MS analyses of this PLFE fraction showed that this fraction was composed of several different tetraether lipids. To better interpret the results of the current experiment, it was necessary to try to further purify this tetraether mixture. The complete purification procedure involved a protection/deprotection sequence and IPL purification by HPLC, and will be described in detail elsewhere (Schaeffer et al. in preparation) (Figure S4).

S1.3. Core Lipid Characterization

Aliquots of the isolated di-or tetraether polar lipids were hydrolyzed in Pyrex™ tubes with PTFE caps. Polar head groups were removed using acid methanolysis (1.2 N HCl in MeOH) at 90 °C for 3 h). Once back to room temperature, the solvent, and excess reagent were removed under reduced pressure, MeOH being added several times to facilitate the removal of HCl and water. The dried crude mixture was extracted using n-C7/IPA (99:1, v/v) to recover the core lipids. In the case of the diether lipids from *P. furiosus*, the composition of the sample was controlled by HPLC-MS using the same binary pump module and ion trap mass spectrometers as described above for polar lipid analysis. The following conditions were used: HPLC column: Zorbax Sil (4.6 x 250 mm, 0.4 ml/min) connected to a pre-column with the same stationary phase, mobile phase: n-C7/IPA 95:5 (isocratic elution mode), MS source: atmospheric pressure chemical ionization source (APCI) used in the positive mode. Conditions for MS analyses

were: nebulizer pressure 43.5 psi, APCI temperature 420 °C, drying temperature 350 °C, drying gas (N_2) flow 5 L min^{-1}, capillary voltage -2 kV, corona 4 mA, scan range *m*/*z* 500–2000 (Esquire 3000[Plus]) or *m*/*z* 500-3000 (HCT). The sole core lipid detected was dialkylglycerol diether (DGD).

The same analytical procedure was used for the analysis of the core lipids from *S. acidocaldarius*, except that a Zorbax RX-Sil narrow bore HPLC column (2.1 x 150 mm, 5mm; Agilent) and a mixture of n-C7/IPA (98.5:1.5 v/v) as mobile phase (isocratic elution) with a flow rate of 0.2 ml/min were used. The core lipids detected comprised glycerol dialkylglycerol tetraethers (GDGT) and calditol-GDGT having 0 to 6 cyclopentane rings.

S1.4. Polar Head Groups Characterization

The polar crude mixture obtained upon acidic hydrolysis of isolated polar lipids (i.e., the part that was not dissolved in *n*-C_7/IPA 99:1 v/v) was transferred into a 3-ml vial using a dichloromethane (DCM)/MeOH (1:1 v/v) mixture, and the solvent was removed under a stream of argon. After the addition of ethyl acetate (EtOAc) (ca. 1 mL), the crude mixture was acetylated using 200 µl of Ac_2O and 100 µl of N-Me-imidazole. After 1 h at room temperature, ca. 2 mL of distilled water containing 25% $CuSO_4$ (v/w) were added, and the mixture was vigorously shacked. The supernatant (i.e., the EtOAc phase) containing the acetylated methoxy-sugars was recovered using a Pasteur pipette and analyzed by GC-FID.

GC analyses of the acetylated methoxy-sugars released by acid hydrolysis were carried out on a Hewlett Packard 6890 gas chromatograph equipped with an on-column injector used in the "track-oven" mode, a flame ionization detector, and an HP-5 fused silica capillary column (30 m x 0.25 mm; 0.25 µm film thickness). H_2 was used as carrier gas (constant flow mode, 2.5 mL.min^{-1}), and the oven was programmed as follows: 70–200 °C (4 °C.min^{-1}), 200–300 °C (10 °C.min^{-1}), isothermal at 300 °C. The different acetylated methoxy-sugars formed upon methanolysis/acetylation were identified by comparing their retention times in GC with those from reference compounds.

Table S1. Recapitulative table of all the tested experimental conditions. D= diether sample, T= tetraether sample, D/T= mixture of diether and tetraether sample at different molar ratios (1:1) or (2:1). M = sample measured in this condition. NM = sample not measured in this condition. D sample 80 % RH: sample not analyzed because only 1 or 2 Bragg peaks were obtained in this condition, not enough to make the calculation.

Samples	Membrane structure	Sample composition (mg)		Tested contrast		Tested temperatures				Tested humidity ratio	
		Diether	Tetraether	100 % D_2O	8 % D_2O	60 °C	70 °C	80 °C	90 °C	80 % RH	95 % RH
**T**	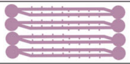	0	3	M	M	M	M	M	NM	M	M
**D**	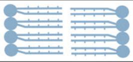	6	0	M	M	M	M	M	NM	M BUT not analysed	M
**D/T(2:1)**	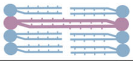	3	3	M	M	M	M	M	M	M	M
**D/T(1:1)**	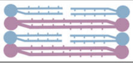	1.5	3	M	M	M	M	M	NM	M	M

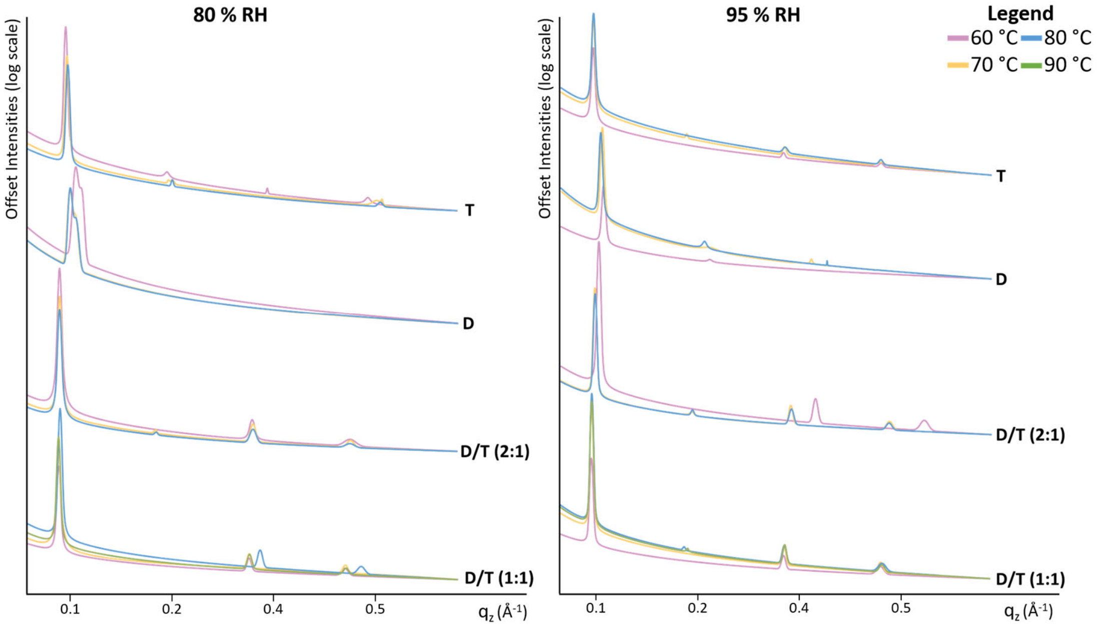

Figure S1. Neutron diffraction of multilayer samples with varying concentrations of purified tetraether (T) and diether lipids (D). **Left:** 1D integrated intensity along the Z direction of the different lipid samples highlighting Bragg peak intensities, run at 80 % RH and 8 % D_2O at different temperatures. **Right:** 1D integrated intensity along the Z direction of the different lipid samples highlighting Bragg peak intensities, run at 95 % RH and 8 % D_2O at different temperatures. D= diether sample, T=tetraether sample, D/T=mixture of diether and tetraether at different molar ratios (1:1) or (2:1).

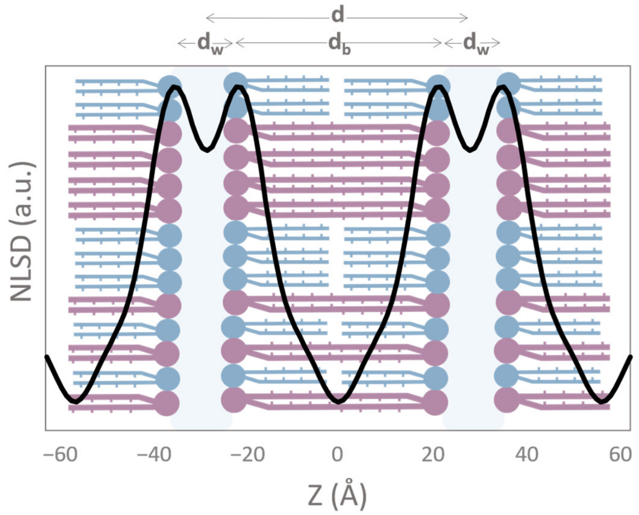

Figure S2. Graphic example of Neutron Scattering Length Density (NSLD) profiles. The bilayer thickness d_b corresponds to the center-to-center distance between headgroups as described in the methods. The water layer thickness d_w is calculated with d-d_b, d being the d-spacing. D/T (1:1) 8 % D_2O at 95 % RH, 70 °C.

Table S2. Recapitulative table of all measurements for all tested samples. The bilayer thickness d_b corresponds to the center-to-center distance between headgroups as described in the methods. The water layer thickness d_w is calculated with d-d_b, d being the d-spacing. D= diether sample, T=tetraether sample, D/T=mixture of diether and tetraether sample at different molar ratios (1:1) or (2:1). NM= not-measure.

Temperature (°C)	d-spacing (Å)							
	D 80 % RH	D 95 % RH	T 80 % RH	T 95 % RH	D/T (2:1) 80 % RH	D/T (2:1) 95 % RH	D/T (1:1) 80 % RH	D/T (1:1) 95 % RH
60	49.4	50.0	51.5	55.5	55.0	54.3	55.6	55.3
70	49.9	50.8	51.5	55.3	54.9	54.2	55.7	55.3
80	49.8	NM	51.0	55.5	55.0	54.3	55.6	54.9
90	NM	NM	NM	NM	NM	NM	55.0	55.1
**Temperature (°C)**	**d_b (Å)**							
	D 80 % RH	D 95 % RH	T 80 % RH	T 95 % RH	D/T (2:1) 80 % RH	D/T (2:1) 95 % RH	D/T (1:1) 80 % RH	D/T (1:1) 95 % RH
60	NM	39	40	44	43	42	44	43
70	NM	36	41	42	42	41	44	43
80	NM	NM	41	44	43	41	44	42
90	NM	NM	NM	NM	NM	NM	43	42
**Temperature (°C)**	**d_w (Å)**							
	D 80 % RH	D 95 % RH	T 80 % RH	T 95 % RH	D/T (2:1) 80 % RH	D/T (2:1) 95 % RH	D/T (1:1) 80 % RH	D/T (1:1) 95 % RH
60	NM	10	12	11	12	12	12	13
70	NM	14	10	13	12	13	12	13
80	NM	NM	10	12	12	13	12	13
90	NM	NM	NM	NM	NM	NM	13	13

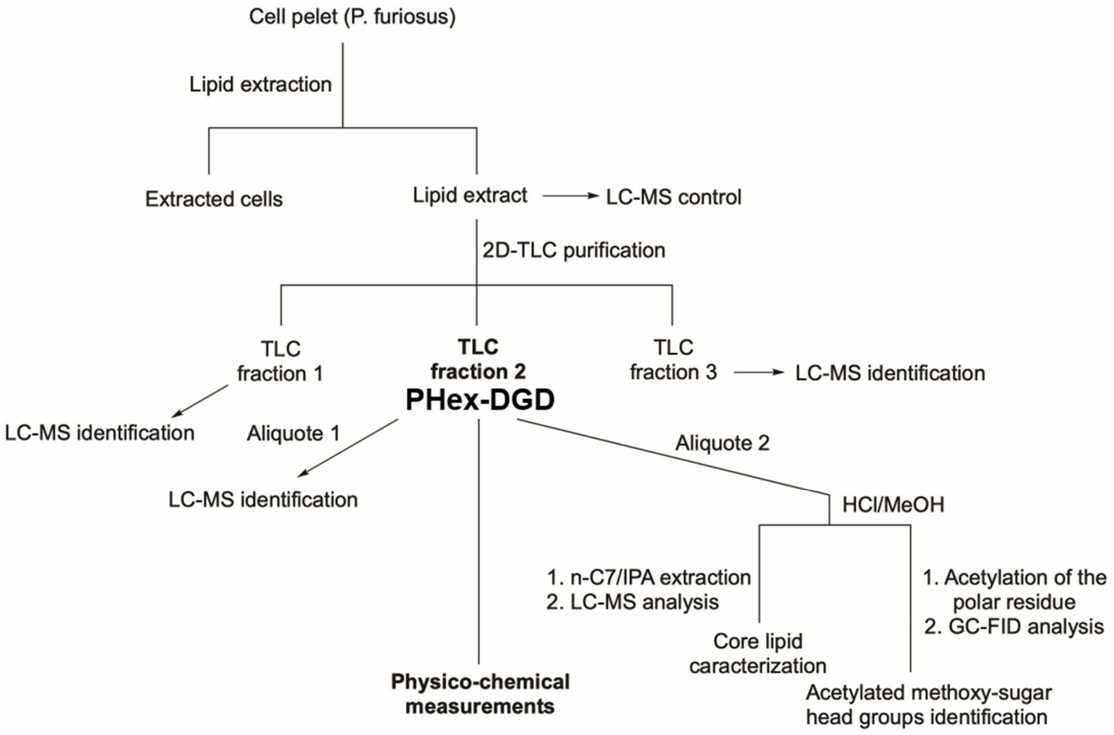

Figure S3. Schematic representation of the diether lipid purification protocol.

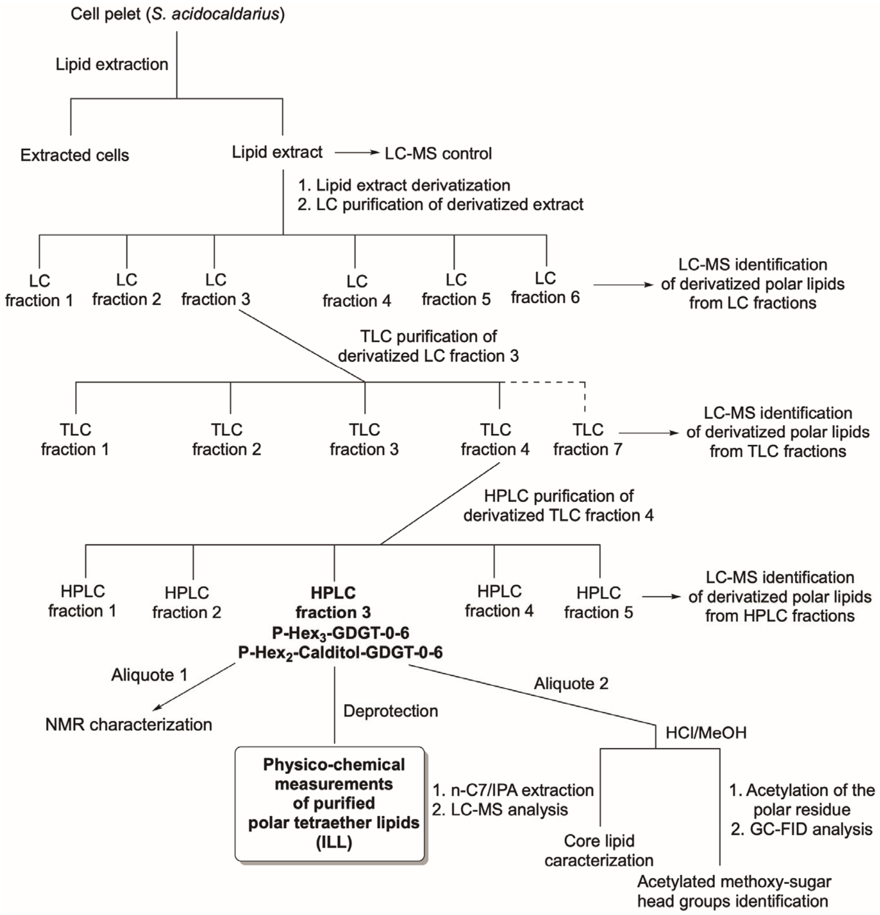

Figure S4. Schematic representation of the tetraether lipid purification protocol.
